# Implications of skill-mix change in general practice: secondary analysis of data from the GP Patient Survey

**DOI:** 10.3399/BJGP.2025.0360

**Published:** 2026-02-24

**Authors:** Charlotte AM Paddison, Theo Georghiou

**Affiliations:** 1 Nuffield Trust, London, UK

**Keywords:** confidence, general practice, patient experience, primary health care, skill mix, trust

## Abstract

**Background:**

The shift in policy and practice towards multiprofessional team working has potential implications for patients’ experience.

**Aim:**

To examine the relationship between delivery of care and patient-reported confidence and trust, and perception of needs met.

**Design and setting:**

Secondary analysis of data from the GP Patient Survey (2023), including 759 149 responders from 6418 English general practices.

**Method:**

Multivariable logistic regression was used to investigate the association between primary outcomes (confidence and trust, and perception of needs met) and patient characteristics, as well as to explore relationships with different combinations of type of health professional and mode of appointment.

**Results:**

When patients are uncertain what type of health professional their appointment was with, odds of expressing confidence and trust decreased by 50%–80% compared with those who saw a GP at their practice. A 50% decrease in the odds of reporting confidence and trust was associated with speaking to a health professional on the phone or by video (adjusted odds ratio [aOR] 0.56, 95% confidence interval [CI] = 0.55 to 0.57 and aOR 0.48, 95% CI = 0.42 to 0.55, respectively), compared with patients whose appointment was at their general practice in person. Similar trends were observed for patient-reported needs met.

**Conclusion:**

Patients who were confused who their general practice appointment was with experienced lower trust and were more likely to perceive that their needs were not met. The impact was magnified when the consultation was remote (phone, video, or message). Helping patients understand new roles and providing clarity on who the patient is seeing are essential for building patient confidence in new models of care.

## How this fits in

To the authors’ knowledge, no previous studies have investigated the impact on patient trust or perception of needs met when patients are unsure what type of health professional they have seen. Using data from a large national survey, this study found that patients expressed lower confidence and trust, and were less likely to report their needs were met in general practice consultations when they were not sure who their appointment was with. The results are novel in demonstrating that the combination of not knowing who you saw and a remote appointment is particularly problematic for patient trust.

## Introduction

Patients’ trust and confidence in health professionals are critical to good-quality care and effective partnerships with patients.^1,2^ High trust relationships are characterised by a perception that health professionals are competent, caring, and have their patients’ best interests at heart,^3^ and can be facilitated by delivery models that enable patients to see the same doctor over time.^4,5^ Previous research has shown patient trust is highest for appointments in person with a nurse and reduces when expectations to see a GP are not met,^6^ and that trust in GPs is higher among older patients and among those of White ethnicity.^2,7^

The value of trust is not, however, limited to its intrinsic significance in giving meaning and substance to therapeutic relationships: trust also has instrumental value.^1^ Trust supports open communication of information and improved adherence to medical advice.^8^ In the context of general practice, specifically, trust and confidence have an important role in supporting the effective management of ambiguous symptoms and undifferentiated illness.^9^ With around a quarter of appointments in general practice in England for medically unexplained symptoms,^10^ trust can play an important role in demedicalising symptoms and reducing onward referral for further investigation or treatment where this is not needed. By avoiding harms because of overdiagnosis and overtreatment this benefits patients, while also managing demand for further investigative tests and referrals to secondary care, which contributes to efficiency within the NHS.

Over a third of patients attending appointments at their general practice in England report their needs are not fully met.^11^ Evidence shows unmet need is higher among people with long-term conditions^11^ and those living in deprived areas — but general practices in these areas are also less able to deal with patients’ needs because of underresourcing relative to affluent areas.^12,13^

Expansion of the primary care multidisciplinary team (MDT) has been proposed to help general practices better meet patients’ needs, while also reducing GP workload,^14^ and address the shortage of GPs.^15^ A majority of patients surveyed report positive experiences with MDT staff,^16^ yet qualitative research also reveals many patients — particularly those in deprived areas or with multiple complex problems — prefer to see a known GP for health concerns that they consider very worrying and potentially serious.^14^ Evidence shows that perceptions of needs having been met is affected not only by who the patients sees (type of professional) but also by the mode of consultation, with video consultations resulting in lower fulfilment of patients’ needs than in-person consultations,^17,18^ and face-to-face consultations more strongly preferred by older patients and those from deprived areas.^14^

Following introduction in 2019 of the Additional Roles Reimbursement Scheme (ARRS),^19^ the staff mix in general practice in England has changed radically. Over 40 000 additional staff have been employed in primary care in a range of roles, including clinical pharmacists, physiotherapists, social prescribing link workers, physician associates, and paramedics;^20^ when including direct patient care staff employed in primary care networks and in general practices, this represents a per-patient rise of 387% over a 9-year period.^21^ Evidence shows that policy to rebalance skill mix does not, however, guarantee better patient experience, higher standards of care, or improved cost-effectiveness.^22^ Experiences from England also provide some salutary lessons about the additional supervision workload for general practice and concerns about safe practice.^23^

Whether small increases in patient satisfaction associated with changes to skill mix represent value for money has been questioned.^24^ In the context of relatively new and contested roles, staff concerns, and potential unmeasured differences in quality of care, this is an area of increasingly robust professional debate.^25^ Potential uncertainty related to skill-mix change is also an issue of concern to the public,^26^ with high-profile individual cases highlighting confusion among the patient and their family about who the patient’s appointment was with leading up to the patient’s death.^27^ A government-ordered review^26^ highlights a concern that patients may be misled, leading to risks to patients’ safety, with coroner reports warning that the term ‘physician associate’ was misleading to the public.

Evidence from England’s GP Patient Survey shows the number of patients who report their last appointment in general practice was with a GP dropped by six percentage points between 2018 (71%) and 2024 (65%), illustrating the impact of skill-mix change on patients’ experience of care.^28^ Results from this survey also show an increase over time in the number of responders who are unsure who their last appointment was with: this figure has more than doubled in 6 years (1.9% in 2018 versus 5% in 2024). Recent evidence also points to a growing loss of confidence in general practice in England; data from the British Social Attitudes survey shows public satisfaction with care provided by general practices is now at just 31%, the lowest level historically recorded.^29^ At present it is unclear how changes in skill mix may be affecting patient trust and confidence, both within consultations and in general practices more broadly.

This study explored the relationship between delivery of care at general practices in England (by type of health professional and mode of appointment) and two aspects of patient-reported experience: trust and confidence, and perceptions of needs met, using data from a large national patient survey.

## Method

Data were analysed from the 2023 GP Patient Survey.^28^ This included 2.65 million questionnaires sent by post in January 2023 to patients aged ≥16 years registered with a general practice in England continuously for ≥6 months, with an option for online completion (see Technical Annex from the survey^30^). The response rate was 28.6%.

Responders reported their gender, age (10 categories), ethnicity (19 categories), sexuality, and any long-term health conditions (yes/no), including long COVID. Socioeconomic deprivation (recorded in national quintiles) was derived from patients’ postcodes.

Survey questions asked patients who their last general practice appointment was with (six response options: GP; nurse; general practice pharmacist; mental health professional; another healthcare professional; and don’t know/not sure who I saw) and what type of appointment it was (six response options: to speak to someone on the phone; to see someone at my general practice; to see someone at another general practice location; to speak to someone on a video call; for a home visit; and to message someone online or by text message).

The GP Patient Survey also asked whether, at their last appointment, the patient had confidence and trust in the healthcare professional they saw or spoke to, and whether their needs were met. The same four response options were available for each of these two questions: ‘yes, definitely’; ‘yes, to some extent’; ‘no, not at all’; and ‘don’t know/can’t say’. For the main analyses, these responses were dichotomised into ‘yes’ (either ‘yes, definitely’ or ‘yes, to some extent’) and ‘no, not at all’ to promote parsimony and interpretability of results.

Type of healthcare professional was recoded into four categories, with mental health professional, pharmacist, and other healthcare professionals combined into a single category, ‘other direct care professional’. Ten age categories were also regrouped into five categories, and 19 ethnic codes into five groupings.

In tables showing the distribution of question responses, individual patient responses were multiplied by survey weights computed to correct for the sampling design and to reduce the impact of non-response bias.^30^

Mixed-effect binary logistic regressions were used to model the relative odds of patients reporting confidence and trust in the healthcare professional they saw or spoke to and, separately, reporting that their needs were met.

For each regression model, data were only included for responders who provided outcome variable responses mapped to the dichotomised ‘yes’ or ‘no’ categories, and who also had complete data on patient characteristic variables (age, gender, ethnicity, sociodeprivation, and long-term condition), type of last appointment, and healthcare professional seen or spoken to.

A series of individual (unadjusted) models were run for each fixed effect (type of healthcare professional, type of appointment, age, gender, ethnicity, quintile of socioeconomic deprivation, and presence of a long-term health condition). A set of models that adjusted for all fixed effects were then run. Finally, in a variant of the adjusted models, type of healthcare professional and type of appointment were interacted together (resulting in 24 combined professional/type categories, for example, ‘GP and seen in person’ and ‘nurse and home visit’). The general practice was included as a random effect.

Model outputs are presented as odds ratios (ORs) and adjusted odds ratios (aORs), with 95% confidence intervals (CIs). Data preparation and analyses were performed in SAS (version 9.4 for Windows).

## Results

In total, 759 149 patients from 6418 English general practices responded to the patient survey. For the majority of responders (66.7%), their last appointment was to see someone at their general practice, followed by speaking to someone on the phone (28.4%) (see Table 1). Only a very small minority, <1% of all patient appointments, were provided by video call (0.6%), home visit (0.4%), or online or text message (0.6%). Just under two in every three patients (61.9%) reported that their last appointment was with a GP.

**Table 1.** Patients’ experience of attending their last appointment at general practices in England^a,b^

Question and answer	*n* (%)
What type of appointment was your last general practice appointment?	
Speak to someone on the phone	197 392 (28.4)
See someone at my general practice	462 968 (66.7)
See someone at another general practice location	22 399 (3.2)
Speak to someone on a video call	4419 (0.6)
Home visit	3076 (0.4)
To message someone online or by text message	3997 (0.6)
Total	694 252
Who was your last general practice appointment with?	
A GP	431 080 (61.9)
A nurse	186 173 (26.7)
A general practice pharmacist	8542 (1.2)
A mental health professional	4006 (0.6)
Another health professional	35 280 (5.1)
Don’t know/not sure who I saw	31 041 (4.5)
Total	696 121

^a^Data show weighted sums and percentages calculated using survey design and non-response weights (by age, gender, geographic location, and general practice; for full details see the Technical Annex).^30^ ^b^Counts are presented rounded to nearest integer, and so sums of counts and the corresponding totals may not appear to be equal.

Although the large majority (64.4%) of patients reported definitely having confidence and trust in the health professional they saw or spoke to at their last general practice appointment, this varied by patient characteristics (Table 2). Similar proportions of males (65.4%) and females (64.2%) reported definite confidence and trust; however, this was lower for patients who identified as non-binary (52.7%). Definite confidence and trust was higher among patients who were older (aged ≥65 years [72.3%] versus aged <25 years [57.0%]), from White ethnic backgrounds (White [66.4%] versus Asian/Asian British/Chinese [53.1%]), and living in affluent areas (least deprived [69.2%] versus most deprived [59.2%] quintile). It also varied by health status, and was lowest for those with an illness limiting day-to-day activities (59.5%) and for those with long COVID (57.6%).

**Table 2.** Sociodemographic characteristics and percentage of subgroup reporting confidence and trust in the health professional at their last general practice appointment^a^

			Did you have confidence and trust in the health professional you saw?, % of subgroup		
**Characteristic**	**Patients,** ***n*** **(** ***N*** **= 677 629)** **677 629**	**%**	**No, not at all** **(*n* = 47 303, 7.0%)**	**Yes, to some extent** **(*n* = 193 792, 28.6%)**	**Yes, definitely** **(*n* = 436 535, 64.4%)**
Gender	671 450				
Female	353 807	52.7	6.9	28.9	64.2
Male	306 662	45.7	6.7	28.0	65.4
Non-binary	2393	0.4	14.4	32.9	52.7
Prefer self-describe/not to say	8588	1.3	17.2	34.9	47.9
Sexuality	669 359				
Heterosexual or straight	596 131	89.1	6.5	28.2	65.3
Gay or lesbian	15 429	2.3	9.4	28.6	62.0
Bisexual	14 136	2.1	9.8	31.0	59.2
Other/prefer not to say	43 663	6.5	11.8	32.8	55.3
Ethnicity	671 524				
White	546 216	81.3	6.4	27.2	66.4
Mixed or multiple ethnic groups	13 746	2.0	10.1	31.4	58.5
Asian or Asian British or Chinese	67 287	10.0	10.0	36.9	53.1
Black, Black British, Caribbean or African	29 165	4.3	6.6	31.4	62.1
Other ethnic group	15 110	2.3	12.3	33.5	54.3
Age, years	674 308				
<25	58 140	8.6	8.9	34.1	57.0
25–44	227 342	33.7	9.6	31.4	59.0
45–64	234 194	34.7	6.3	27.3	66.5
65–84	137 256	20.4	3.4	24.3	72.3
≥85	17 376	2.6	3.0	24.7	72.3
Deprivation quintile	677 293				
1 (least deprived)	130 681	19.3	4.9	25.9	69.2
2	133 138	19.7	5.6	27.1	67.3
3	135 967	20.1	6.5	28.3	65.2
4	139 321	20.6	8.1	30.3	61.6
5 (most deprived)	138 186	20.4	9.6	31.2	59.2
Long-term condition	630 683				
Yes	374 947	59.5	7.3	28.3	64.4
No	255 736	40.5	5.8	28.0	66.2
Illness limiting day-to-day activities	361 182				
Yes	221 257	61.3	9.4	31.1	59.5
No	139 925	38.7	4.6	24.4	71.0
Long COVID	602 503				
Yes	34 433	5.7	10.7	31.6	57.6
No	568 070	94.3	6.3	27.5	66.2

^a^Data show weighted percentages calculated using survey design and non-response weights (by age, gender, geographic location, and general practice; for full details see the Technical Annex).^30^ Data include all patients who gave a valid response to question on confidence and trust; it excludes multicoded or missing responses and those who reported ‘don’t know/can’t say’.

Although the overall majority of patients had definite confidence and trust in health professionals delivering their care (64.4%), or did to some extent (28.6%), a substantive minority did not at all (7.0%) (Table 2).

Almost one in 10 patients (9%) reported that their needs were ‘not at all’ met at their last general practice appointment. Patients in areas of high deprivation, those with an illness limiting their day-to-day activities, or with long COVID were more likely than others to report that their needs were not met (12%, 12%, and 14%, respectively). For needs met, patterns of those who answered ‘yes, definitely’ with respect to different patient characteristics were broadly similar to those described above for confidence and trust (see Supplementary Table S1).

Relative odds of patients reporting confidence and trust in their healthcare professional, and that their needs were met, are shown in Tables 3 and 4, respectively (see also Supplementary Figures S1 and S2).

**Table 3.** Results of regression analyses showing associations between patient-reported confidence and trust in care at last general practice consultation and type of health professional, type of appointment, and patient characteristic^a^

Characteristic	OR (95% CI)	Adjusted OR^b^ (95% CI)
Type of health professional		
GP	Reference	Reference
Nurse	1.61 (1.56 to 1.65)	1.33 (1.29 to 1.37)
Other direct care professional	0.68 (0.65 to 0.70)	0.67 (0.64 to 0.69)
Don’t know/not sure who I saw	0.37 (0.35 to 0.39)	0.45 (0.42 to 0.47)
Type of appointment		
Seen at my general practice	Reference	Reference
Spoke on phone	0.49 (0.48 to 0.50)	0.56 (0.55 to 0.57)
Spoke on a video call	0.39 (0.34 to 0.44)	0.48 (0.42 to 0.55)
Seen at another general practice location	0.72 (0.68 to 0.77)	0.85 (0.79 to 0.91)
Home visit	0.69 (0.59 to 0.79)	0.53 (0.46 to 0.62)
Message online or by text message	0.31 (0.27 to 0.35)	0.43 (0.38 to 0.49)
Gender		
Female	Reference	Reference
Male	1.14 (1.12 to 1.17)	1.04 (1.02 to 1.06)
Non-binary or prefer to self-describe/not say	0.42 (0.39 to 0.45)	0.54 (0.50 to 0.58)
Ethnicity		
White	Reference	Reference
Mixed or multiple ethnic groups	0.59 (0.55 to 0.64)	0.81 (0.75 to 0.88)
Asian, Asian British or Chinese	0.66 (0.64 to 0.69)	0.85 (0.81 to 0.88)
Black, Black British, Caribbean or African	1.08 (1.02 to 1.15)	1.32 (1.24 to 1.40)
Other ethnic group	0.52 (0.49 to 0.56)	0.70 (0.66 to 0.75)
Age, years		
<25	0.63 (0.59 to 0.66)	0.66 (0.63 to 0.70)
25–44	0.62 (0.60 to 0.63)	0.61 (0.60 to 0.63)
45–64	Reference	Reference
65–84	1.77 (1.72 to 1.82)	1.78 (1.72 to 1.83)
≥85	2.17 (1.99 to 2.36)	2.33 (2.14 to 2.55)
Deprivation quintile		
1 (lowest deprivation)	Reference	Reference
2	0.88 (0.84 to 0.92)	0.90 (0.86 to 0.94)
3	0.77 (0.74 to 0.80)	0.82 (0.79 to 0.86)
4	0.65 (0.63 to 0.68)	0.73 (0.70 to 0.76)
5 (highest deprivation)	0.56 (0.54 to 0.58)	0.66 (0.63 to 0.69)
Long-term condition (including long COVID)		
Yes	0.82 (0.80 to 0.84)	0.64 (0.62 to 0.65)
No	Reference	Reference

^a^Only those patients with complete information for all covariates are included (*N *= 571 362). ORs <1 indicate patient subgroup less likely to report confidence and trust than reference category. *P*<0.001 for all except ethnicity subgroup ‘Black, Black British, Caribbean or African’, where unadjusted OR *P *= 0.007. ^b^Adjusted for age, gender, ethnicity, deprivation, presence of a longstanding health condition (fixed effects), and practice (random effect). OR = odds ratio.

**Table 4.** Results of regression analyses showing the associations between patient-reported needs having been met in their last general practice consultation and the type of health professional, type of appointment, and patient characteristic^a^

Characteristic	OR (95% CI)	Adjusted OR^b^ (95% CI)
Type of health professional		
GP	Reference	Reference
Nurse	1.76 (1.72 to 1.81)	1.45 (1.41 to 1.49)
Other direct care professional	0.69 (0.67 to 0.72)	0.68 (0.66 to 0.70)
Don’t know/not sure who I saw	0.39 (0.37 to 0.40)	0.47 (0.45 to 0.49)
Type of appointment		
Seen at my general practice	Reference	Reference
Spoke on phone	0.50 (0.49 to 0.51)	0.57 (0.56 to 0.58)
Spoke on a video call	0.36 (0.32 to 0.40)	0.44 (0.40 to 0.50)
Seen at another general practice location	0.66 (0.62 to 0.70)	0.77 (0.73 to 0.82)
Home visit	0.74 (0.65 to 0.85)	0.58 (0.51 to 0.67)
Message online or by text message	0.34 (0.30 to 0.38)	0.48 (0.43 to 0.54)
Gender		
Female	Reference	Reference
Male	1.06 (1.03 to 1.08)	0.96 (0.94 to 0.98)
Non-binary or prefer to self-describe/not say	0.45 (0.42 to 0.48)	0.59 (0.55 to 0.63)
Ethnicity		
White	Reference	Reference
Mixed or multiple ethnic groups	0.56 (0.53 to 0.60)	0.78 (0.73 to 0.83)
Asian, Asian British or Chinese	0.60 (0.58 to 0.62)	0.77 (0.74 to 0.79)
Black, Black British, Caribbean or African	0.84 (0.80 to 0.89)	1.03 (0.98 to 1.08)
Other ethnic group	0.49 (0.46 to 0.52)	0.65 (0.61 to 0.69)
Age, years		
<25	0.54 (0.52 to 0.57)	0.58 (0.55 to 0.60)
25–44	0.61 (0.59 to 0.62)	0.61 (0.59 to 0.62)
45–64	Reference	Reference
65–84	1.81 (1.77 to 1.86)	1.79 (1.75 to 1.84)
≥85	1.90 (1.77 to 2.04)	1.98 (1.84 to 2.13)
Deprivation quintile		
1 (lowest deprivation)	Reference	Reference
2	0.89 (0.86 to 0.93)	0.92 (0.88 to 0.95)
3	0.80 (0.78 to 0.83)	0.86 (0.83 to 0.89)
4	0.69 (0.67 to 0.72)	0.79 (0.76 to 0.82)
5 (highest deprivation)	0.60 (0.58 to 0.62)	0.72 (0.69 to 0.74)
Long-term condition (including long COVID)		
Yes	0.88 (0.86 to 0.90)	0.67 (0.65 to 0.68)
No	Reference	Reference

^a^Only those patients with complete information for all covariates are included (*N *= 572 849). ORs <1 indicate patient subgroup less likely to report needs met than reference category. *P*<0.0001 for all except ethnicity subgroup ‘Black, Black British, Caribbean or African’, where adjusted OR *P *= 0.3. ^b^Adjusted for age, gender, ethnicity, deprivation, presence of a longstanding health condition (fixed effects), and practice (random effect). OR = odds ratio.

When compared with patients who saw or spoke to a GP, those whose last appointment was with a nurse were more likely to report confidence and trust (aOR 1.33, 95% CI = 1.29 to 1.37) and that their needs were met (aOR 1.45, 95% CI = 1.41 to 1.49), whereas for patients who saw a direct care professional such as a pharmacist or mental health professional at their general practice, this trend reversed (aOR 0.67, 95% CI = 0.64 to 0.69 and aOR 0.68, 95% CI = 0.66 to 0.70 for confidence and trust, and needs met, respectively) (Tables 3 and 4). A 50% decrease in the odds of reporting confidence and trust was associated with speaking to a health professional on the phone or by video (aOR 0.56, 95% CI = 0.55 to 0.57 and aOR 0.48, 95% CI = 0.42 to 0.55, respectively), compared with patients whose last appointment took place at their general practice in person (Table 3).

**Figure 1.**
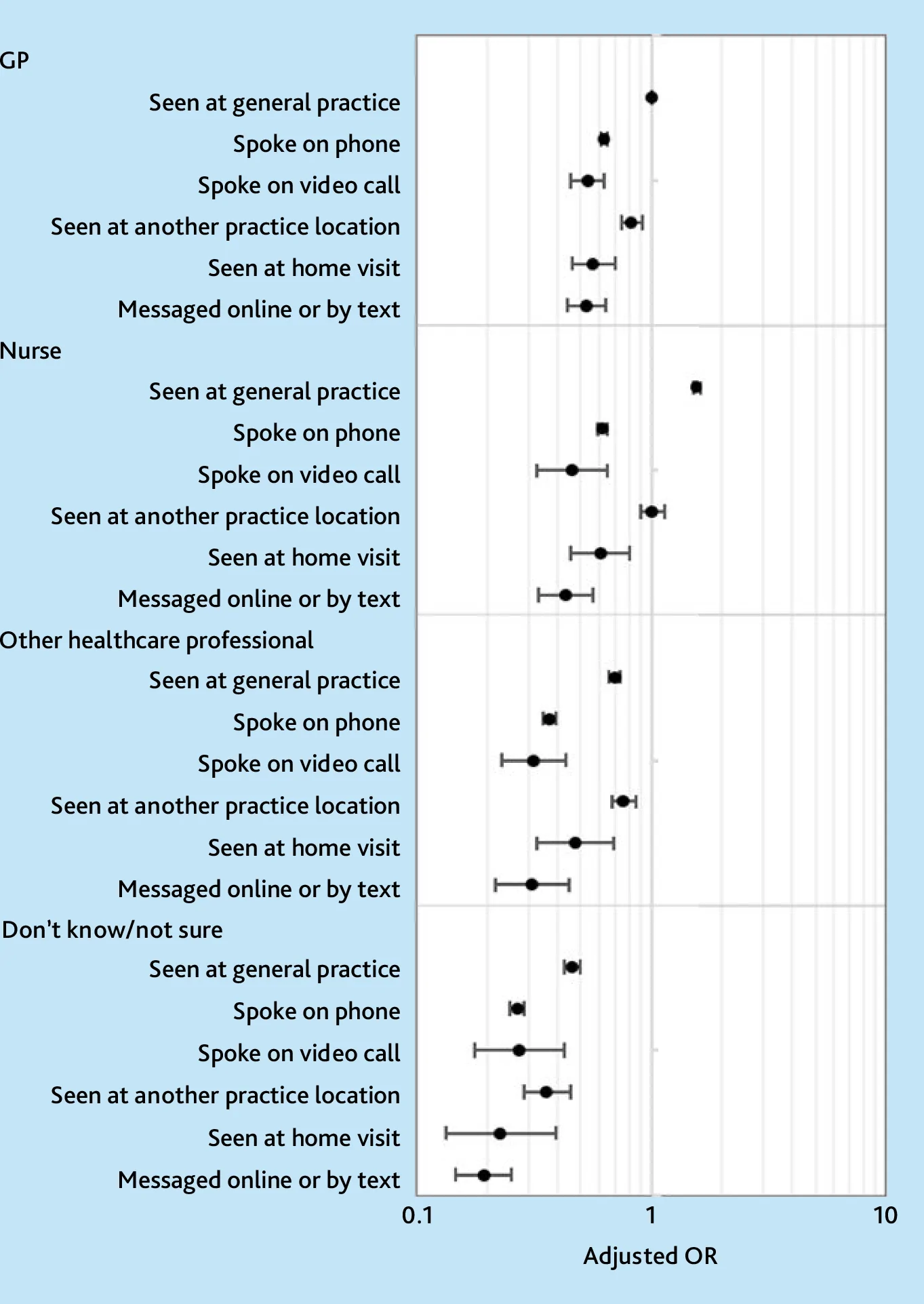
Results of regression analyses examining the variation in patient-rated confidence and trust in health professional at their last general practice consultation, by the type of health professional and type of appointment combined. ORs <1 indicate subgroup less likely to report confidence and trust than reference category (which is seeing a GP in person at the general practice). Data for this figure are provided in Supplementary Table S2. OR = odds ratio.

ORs from interaction models are displayed in Figure 1 (data shown in Supplementary Table S2). These show the likelihood of expressing confidence and trust for different combinations of healthcare professional and type of appointment. Patients reported the highest levels of confidence and trust when their last appointment was with a nurse and in person at their general practice (aOR 1.57, 95% CI = 1.51 to 1.62). A consistent trend showing that patient trust was lower when appointments were by phone, video, or by message, when compared with appointments delivered in person at the patients’ general practice, was observed across all health professional groups.

Patients who were not sure what type of health professional they saw or spoke to at their last appointment reported significantly lower trust and confidence, across all six appointment modalities in this study (Figure 1).

Compared with patients who reported they saw a GP in person at their practice, for patients who were not sure about who their appointment was with and whose appointment was by text or online message the odds of expressing confidence and trust decreased by 80% (aOR 0.19, 95% CI = 0.15 to 0.25). For similar patients whose appointment was on the phone (aOR 0.27, 95% CI = 0.25 to 0.29) or who were seen at another general practice location (aOR 0.36, 95% CI = 0.29 to 0.45), the odds of expressing confidence and trust decreased by around 70%. This effect persists for patients seen at their general practice but who did not know what type of professional they saw, albeit in reduced magnitude, with the odds of reporting confidence and trust decreasing by 50% (aOR 0.46, 95% CI = 0.43 to 0.50) compared with patients who reported they saw a GP (Figure 1).

In terms of whether patients reported that their needs were met the magnitude and direction of effects were similar (see Supplementary Table S3 and Figure S3).

## Discussion

### Summary

In a study of 759 149 responders to a national survey in England, it was found that patients expressed lower confidence and trust, and were less likely to report their needs were met in general practice consultations when they were not sure who their appointment was with. The impact on trust of this confusion about professional role was magnified when care was delivered remotely. For patients who were not sure what type of health professional they spoke to in a consultation by phone, video, or message, the likelihood of reporting confidence and trust decreased substantially, with odds reduced by up to 80%, when compared with patients who saw a GP in person at their practice.

Two big changes in general practice have happened simultaneously in the past 5 years: change in skill mix with a shift towards multiprofessional teams and, separately, an increase in appointments delivered remotely. The study’s results are novel in demonstrating how these policy changes intersect, and how they are related to differences in patients’ experience at their general practice. The results showed that the combination of not knowing who you saw and a remote appointment is particularly problematic for patient trust.

In addition to this, almost one in 10 patients in this study reported that their needs were ‘not at all’ met at their last general practice appointment. Concerningly, some patient groups with higher healthcare needs — for example, those living in deprived areas and those with a chronic illness — were more likely to report their needs were not met, and to report lower confidence and trust. This raises questions about the contribution of health care itself to the social patterning of health outcomes, including unanswered questions about the relationship between skill-mix change and the inverse care law in general practice.

### Strengths and limitations

The study has some limitations: certain groups of patients, for example, those triaged to see a mental health professional, could be more likely to have lower baseline trust than patients whose appointment is with a GP or nurse. As the GP Patient Survey does not collect detailed information on all of the 18 different types of health professionals now employed by general practices in England, the current study was unable to make granular distinctions or compare every possible role. Some patients may be uncertain who they saw at their last appointment because they have simply forgotten. The absolute number of patients in some appointment types, for example, home visits or online message, were relatively small at around 3000–4000 responders each; and smaller for combined subgroups, for example, <300 for those who had a video appointment and who did not know who their appointment was with. Nevertheless, the GP Patient Survey is a very large national dataset, with carefully implemented processes for quality assurance and weighting to correct for non-response bias, and the large sample size provided adequate statistical power for the analysis — including for subgroup comparisons. In addition, key observations presented remained substantively similar with alternative regression model construction (including fixed effect only and ordinal models).

### Comparison with existing literature

Using data from a large national survey of patient experience in general practice, the current findings build on previous research showing that trust varies by patient characteristics,^2,7^ and research demonstrating that it is more difficult to establish rapport and build trust when care is delivered remotely.^31^ Evidence shows video consultations result in lower fulfilment of patient needs than in-person consultations,^17^ and the current findings support this. However, the high level of trust and perception of needs met associated with chat visits using synchronous and asynchronous text communication reported in previous research^17^ was not observed in the current study. Although the results in the current study highlight an association between patients’ uncertainty about professional roles and lower confidence and trust, further research is needed to better understand the nature of this uncertainty, how specifically it may have an impact on trust, and, importantly, to identify what actions may be most effective in building confidence in new roles among patients. Seeing someone the patient knows already, for example, may help to mitigate uncertainty about roles and preserve trust even in the context of a remote appointment, in which case practical strategies for promoting continuity could be important in helping general practices to build patient trust and confidence. Although research has shown video appointments can help to build trust and reduce complaints in the context of urgent care,^32^ in the context of general practice, evidence suggests remote consultations can make it more difficult to build rapport and trust with patients.^33^ Although there is some evidence that patients’ perceptions that their needs are met is associated with higher availability of face-to-face appointments with GPs,^18^ further research is needed to better understand the interaction between appointment modality (that is, face to face or remote), trust, and perceptions of needs met.

Very limited evidence is available to assess the proportion of patients who are confused about who they have seen, which, anecdotally, is high.^34^ The current study helps to quantify the extent of this problem using robust data, addressing a gap in evidence by providing national data that show how many patients are unsure who they saw at their last appointment in general practice. The current study found that confusion about the different roles of health professionals working in general practice is likely to affect around one in every 20 patient consultations, with 5% of all patients in the 2024 GP Patient Survey reporting they were unsure which type of health professional they saw or spoke to at their general practice.^28^ In total, across the 373 million standard appointments (excluding COVID-19 vaccinations) delivered by general practices in England each year,^35^ using weighted data, the current study estimates approximately 18 million (5%) patient appointments may be being affected by uncertainty about who the patient saw — to the potential detriment of patients’ trust and confidence in their care.

### Implications for research and practice

Getting easier access to appointments at general practices is the public’s highest healthcare priority, according to polling in 2024,^36^ with policymakers prioritising increasing the availability of appointments at general practices.^37^ The current findings question if these appointments are meeting patients’ needs. Patients living in the most deprived areas, a group that, on average, have higher healthcare needs than those living in areas of lower deprivation,^38^ were more likely to report their needs were not met. As reported in the current study, only one in two people with an illness limiting day-to-day activities reported that their needs were definitely met at their last general practice appointment. International research shows that primary care transformation (of which skill-mix change is one key component) in the context of deprived populations has mixed results, with around half of studies (nine out of 18) showing no change or widening health inequalities.^39^ The current findings, which resonate with research from other countries, should invite general practices, royal colleges, and policy leaders to deeply consider the purpose of general practice and ask a critical question: does the current delivery model in general practice meet the needs of those patients who need care most?

The results also show a non-trivial proportion of patients are confused about who they see and this is associated with lower trust, particularly in remote consultations. One way general practices can help build patient trust, in practical terms, is by ensuring all staff introduce themselves to patients in a way that helps patients to clearly understand their professional role, for example, ‘Hello my name is … and I’m a paramedic/physician associate/pharmacist working in this practice’. New skills will be required to actively build confidence and connection with patients: both those who are physically remote and, given evidence showing a loss of continuity of care in general practice,^40^ those with whom a healthcare professional may not have an ongoing therapeutic relationship.

Further research is needed to unpick the relationships between skill-mix change and patient trust. Uncertainty about what healthcare professional will be seen in general practice is clearly an issue of significant concern to the public and is associated with a loss of social trust in medicine,^3^ as reflected in high-profile media coverage of patient deaths in England.^26,27^ It is possible that skill-mix change may be a potential factor contributing to the erosion of public confidence in general practice evident in recent years.^29^ The study cannot, however, be definitive on that. Although the percentage of patients who report they ‘do not know’ who they saw at their appointment has more than doubled^28^ in the 6-year period since introduction of the ARRS, and these temporal associations indicate some support for this hypothesis, no causal relationship can be established. Further research is needed to test the working hypothesis that rapid introduction of new roles into general practice at scale and pace may create uncertainty for patients, and contribute to a loss of trust.

What is clear is that more effective communication is needed from general practices and from policy at a national level to build public awareness and confidence in new staff roles. In the context of ongoing toxicity in public debate about the role of physician associates working at general practices,^25^ expressions of a lack of confidence from GPs in their allied health professional colleagues,^41^ and a paucity of good-quality evidence,^25^ the loss of patient confidence is understandable and must be addressed. In the context of proposed reform and ongoing changes to the delivery model for general practice,^42^ whether that be skill-mix change, shift to remote consultations, or the implementation of artificial intelligence into general practices, the strategic importance of building patient trust and maintaining public confidence needs to be recognised by both general practices and healthcare leaders.

## Data

Under the terms of the data-sharing agreement, the authors are not able to share the data used for this study. However, the data are available by agreement with NHS England.
